# Cost-effectiveness analysis of the combination of low-dose nivolumab with triple metronomic chemotherapy for advanced head and neck squamous cell carcinoma in China

**DOI:** 10.3389/fonc.2025.1542792

**Published:** 2025-09-01

**Authors:** Yingdan Cao, Fenghao Shi, Xiaoxia Wei, Sheng Han, Yu Fang

**Affiliations:** ^1^ Department of Pharmacy Administration and Clinical Pharmacy, School of Pharmacy, Xi’an Jiaotong University, Xi’an, China; ^2^ Center for Drug Safety and Policy Research, Xi’an Jiaotong University, Xi’an, China; ^3^ International Research Center for Medicinal Administration, Peking University, Beijing, China; ^4^ Department of Pharmacy, Shengli Clinical Medical College of Fujian Medical University, Fujian Provincial Hospital, Fuzhou, China

**Keywords:** cost-effectiveness, low-dose nivolumab, triple metronomic chemotherapy, head and neck squamous cell carcinoma, partitioned survival model

## Abstract

**Background:**

The combination of low-dose nivolumab with triple metronomic chemotherapy (TMC-I) proposes a novel approach, potentially enhancing patient prognosis while mitigating financial barriers. The purpose of this study was to compare the cost-effectiveness of TMC-I compared to triple metronomic chemotherapy (TMC) in advanced head and neck squamous cell carcinoma (HNSCC) patients in China, the largest developing country.

**Methods:**

A partitioned survival model (PSM) was developed based on a randomized clinical trial. Costs and utility were derived from open-access databases and literatures. The primary outcome was incremental cost-effectiveness ratio (ICER). A willingness-to-pay (WTP) threshold of ¥44,679/QALY based on supply-side and ¥134,037/QALY based on demand-side were set. Sensitivity analyses and scenario analysis were conducted; subgroup analyses were also included.

**Results:**

TMC-I yielded an additional 0.41 quality-adjusted life years (QALYs) while increasing costs by ¥47,346.98 relative to TMC, leading an ICER of ¥116,374.22/QALY. In scenario analysis which the utilities calculated by the time-to-death (TTD) were adopted, the results showed that the ICER was ¥114,795.25/QALY. In the probabilistic sensitivity analysis, the probabilities that TMC-I was cost-effective at thresholds of ¥134,037/QALY, ¥44,679/QALY gained were 60.9%, 9.4%, respectively. Subgroup analysis results indicated TMC-I was dominated vs. TMC for patients with no previous taxane and PD-L1 score >50.

**Conclusion:**

For Patients with recurrent or newly diagnosed advanced head and neck squamous cell carcinoma, TMC-I is cost-effective at a WTP thresholds of ¥134,037/QALY and is not cost-effective when the WTP thresholds was ¥44,679/QALY compared with TMC.

## 1 Introduction

Head and neck cancers (HNC) are among the most common malignant cancers (1), with an incidence increasing with age, and most patients being diagnosed between the ages of 50 and 70 (2). In China, the incidence rate of HNC ranks 6th and the mortality rate 7th among men (3). The most prevalent pathological type is squamous cell carcinoma, accounting for approximately 95% of HNC. The incidence of head and neck squamous cell carcinoma (HNSCC) is quite high worldwide, ranking seventh among malignant cancers (4, 5). Tobacco and alcohol are major risk factors for the development of HNSCC.

According to the Chinese Society of Clinical Oncology (CSCO) guidelines: surgery, radiotherapy, and chemotherapy are the traditional treatment options for HNSCC (6). However, challenges persist with traditional treatment options, including low overall survival rates, limited curative potential, and a high tendency for local recurrence or distant metastasis. Approximately 40% to 60% of advanced-stage patients may experience relapse or metastasis following treatment, contributing to the relatively low 5-year survival rate of less than 40% (7). Consequently, the prognosis for advanced HNSCC remains poor, and there is still an unmet clinical need for effective treatments.

In recent years, immune checkpoint inhibitors, such as programmed cell death protein-1 (PD-1) monoclonal antibodies, have shown promising results in the treatment of advanced HNSCC and have been recommended by international guidelines (8, 9). The CHECKMATE-141 trial (10) demonstrated that nivolumab significantly improves the prognosis of HNSCC patients. In 2016, the U.S. Food and Drug Administration (FDA) approved nivolumab for this indication, followed by its approval in China in October 2019. However, the high cost of nivolumab poses a significant economic burden on patients. Additionally, some scholars have evaluated the cost-effectiveness of nivolumab compared to standard treatments, and the results consistently indicate that these nivolumab is not cost-effective (11–13). In low- and middle-income countries, only 1%-3% of recurrent or metastatic head and neck squamous cell carcinoma patients can afford immune checkpoint inhibitors (14).

A phase III clinical trial (14) investigated whether adding low-dose nivolumab to triple metronomic chemotherapy (TMC-I) could improve the overall survival (OS) in this patient population. The introduction of low-dose of nivolumab significantly improved the 1-year OS from 16.3% (95% Confidence Interval (CI), 8.0 to 27.4) to 43.4% (95% CI, 30.8 to 55.3; hazard ratio, 0.545; 95% CI, 0.362 to 0.820). The median OS in the triple metronomic chemotherapy (TMC) and TMC-I groups was 6.7 months (95% CI, 5.8 to 8.1) and 10.1 months (95% CI, 7.4 to 12.6), respectively. The incidence of grade 3 and higher adverse events was 50% in the TMC group and 46.1% in the TMC-I group. Given its superior efficacy and safety compared to TMC alone, the combination of low-dose nivolumab and TMC presents a potentially new treatment option for patients unable to access full-dose checkpoint inhibitors, leading to improved prognosis. In light of these findings, this study assesses the cost-effectiveness of adding low-dose nivolumab to TMC compared to TMC alone in the treatment of advanced HNSCC from the perspective of Chinese health care system.

## 2 Materials and methods

### 2.1 Model overview

**Figure 1:**
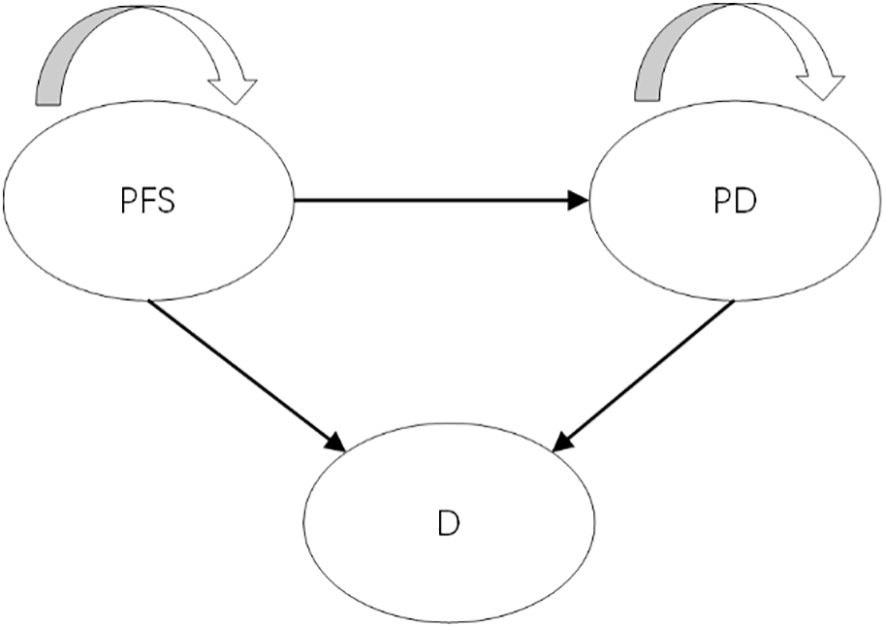
The structure of partitioned survival model. PFS, progression-free survival; PD, progressed disease; D, death.

A partitioned survival model (PSM) was developed to assess incremental costs and quality-adjusted life years (QALYs) for a simulated patient cohort in Microsoft Excel 2019. The model incorporated three mutually exclusive health states: progression-free survival (PFS), progressive disease (PD), and death (**Figure 1**). All patients entered the model in the PFS state, with the possibility of remaining in PFS status, transition to the PD status, or progressing to death. Once patients transitioned from PFS to PD, they could not revert to the PFS state but could continue to progress within PD or move to the death state. The model simulated the cohort until 99% of patients had died. Each cycle had a duration of 3 weeks, which was based on the dosing cycle of nivolumab in the clinical trial. Incremental cost-effectiveness ratios (ICERs) were utilized to assess cost-effectiveness, with a willingness-to-pay (WTP) threshold set as ¥44,679 per QALY gained based on supply-side (0.5 times 2023 Chinese gross domestic product [GDP] per capita) and ¥134,037 per QALY gained based on demand-side(1.5 times 2023 Chinese GDP per capita) (15–17). A discount rate of 5% was applied to both health outcomes and costs (17).

### 2.2 Trial background

Clinical efficacy and safety data for the model were derived from a phase III clinical trial conducted in India (14), which investigated the combination of low-dose nivolumab and triple metronomic chemotherapy in patients with advanced HNSCC. The target patients in the model were adults with recurrent or newly diagnosed advanced HNSCC being treated with palliative intent, and an Eastern Cooperative Oncology Group performance status (ECOG PS) of 0-1. The initial age of the cohort patient was 46 years in TMC arm and 50 years in TMC-I arm. Patients in the clinical trial were randomized to receive either triple metronomic chemotherapy (TMC arm) or TMC in addition of intravenous nivolumab 20 mg (TMC-I arm).

The TMC regimen included oral celecoxib 200 mg twice daily, methotrexate 9 mg/m2 weekly, and oral erlotinib 150 mg once daily. The TMC-I regimen as described above, with the addition of intravenous nivolumab 20 mg once every 3 weeks. Patients continue to receive treatment until intolerable adverse events occur or disease progression. Upon disease progression, systemic therapy was received by 46 patients (61.3%) in the TMC arm and 28 (36.8%) in the TMC-I arm. Best supportive care (BSC) was provided to those who did not receive systemic therapy.

### 2.3 Clinical data and survival curve extrapolation

Because the clinical trial follow-up period did not capture long-term survival, parametric extrapolation was necessary to estimate lifetime progression and survival outcomes, as required for health economic modeling. Probabilities of PFS and overall survival were extracted from the Kaplan–Meier (KM) curves in the clinical trial using GetData Graph Digitizer (http://getdata-graph-digitizer.com), following the method proposed by Guyot et al. (18). The reconstruction of Individual Patient Data (IPD) and extrapolation of survival outcomes were conducted in R Studio. Various parametric distributions, including Weibull, exponential, log-logistic, log-normal, Gompertz, and generalized gamma, were fitted to the reconstructed IPD. Goodness-of-fit was evaluated through visual inspection and the Akaike information criterion (AIC) and Bayesian information criterion (BIC). Lower AIC and BIC values combined with reasonable visual effects indicate a better fit of the selected model (19), When AIC and BIC results were inconsistent, the distribution with the lowest BIC was selected, as BIC applies a stricter penalty for model complexity and is often preferred for long-term survival extrapolation in economic evaluations (20). Besides, the log cumulative hazard plots of TMC arm and TMC-I arm for PFS and OS (see **Supplementary Materials**) were compared in order to appropriately select the survival models. The log cumulative hazard plots of two groups were not parallel, indicating that the proportional hazards assumption may not hold, necessitating separate analyses for each treatment arm.

AIC and BIC values for each distribution, as well as the final selected model, are presented in **Table 1**. The reconstruction of KM curves and the fitting and extrapolation of observational and predicted curve are shown in **Supplementary Materials**. For OS and PFS in the TMC-I group, exponential models were preferred. For TMC, Weibull models provided superior fit. The survival parameters gained in the weibull survival function and the exponential survival function model are provided in **Table 2**.

**Table 1:** Summary of the goodness of statistical fit of the KM curve.

	OS in TMC-I (AIC)	OS in TMC-I (BIC)	PFS in TMC-I (AIC)	PFS in TMC-I (BIC)	OS in TMC (AIC)	OS in TMC (BIC)	PFS in TMC (AIC)	PFS in TMC (BIC)
Exponential	304.37	306.70	342.97	345.30	371.31	373.63	367.31	369.63
Weibull	303.87	308.53	342.85	347.52	354.23	358.86	352.15	356.78
Gamma	304.11	308.77	342.77	347.43	357.29	361.93	353.56	358.19
Log logistic	305.24	309.90	343.18	347.85	358.74	363.37	354.48	359.12
Log normal	308.22	312.88	346.58	351.24	377.68	382.31	372.21	376.84
Gompertz	345.30	347.52	351.24	348.59	355.40	360.03	357.82	362.45
Generalized gamma	373.63	358.86	382.31	360.03	355.73	362.68	354.14	361.10

PFS, progression-free survival; OS, overall survival; AIC, Akaike information criterion; BIC, Bayesian information criterion; TMC, triple metronomic chemotherapy; TMC-I, TMC with intravenous nivolumab.

**Table 2:** Overview of all model parameters.

Variables	Baseline value	Range		Distribution	Reference
		Minimum	Maximum		
Survival					
Exponential OS curve of TMC-I	λ = 0.0680559	–	–	–	(15)
Weibull OS curve of TMC	λ = 8.60491	–	–	–	(15)
	γ = 1.7284	–	–	–	(15)
Exponential PFS curve of TMC-I	λ = 0.102431	–	–	–	(15)
Weibull PFS curve of TMC	λ = 6.07724	–	–	–	(15)
	γ = 1.58831	–	–	–	(15)
Costs ¥					
PD-L1 test cost	344.539	275.631	413.447	Gamma	(24)
Cost of Drug, ¥ per mg					
methotrexate	0.900	0.720	1.900	Gamma	(21)
celecoxib	0.022	0.002	0.029	Gamma	(21)
erlotinib	0.378	0.031	0.594	Gamma	(21)
nivolumab	92.500	92.500	114.669	Gamma	(21)
Cost of Administration, ¥	377.217	301.774	452.661	Gamma	(37)
Cost of Best supportive care, ¥	1,119.575	895.660	1,343.490	Gamma	(25)
Cost of Terminal cancer care, ¥	14,487.694	11,590.155	17,385.232	Gamma	(26)
Cost of Subsequent Therapy, ¥	18,150.465	14,520.372	21,780.557	Gamma	(30)
Cost of AEs, ¥					
Anemia	4,244.580	3,395.664	5,093.496	Gamma	(30)
Neutropenia	3,684.083	2,947.266	4,420.899	Gamma	(30)
Thrombocytopenia	28,353.086	22,682.469	34,023.703	Gamma	(30)
Hypokalemia	192.871	154.297	231.445	Gamma	(28)
Fatigue	921.376	737.101	1,105.651	Gamma	(30)
Rash	39.640	31.712	47.568	Gamma	(27)
Mucositis	33.104	26.483	39.725	Gamma	(29)
Utilities					
utility of PFS (TMC-I)	0.680	0.402	0.900	Beta	(31)
utility of PD (TMC-I)	0.660	0.607	0.680	Beta	(31)
utility of PFS (TMC)	0.610	0.485	0.673	Beta	(31)
utility of PD (TMC)	0.540	0.378	0.645	Beta	(31)
Anemia	0.125	0.100	0.150	Beta	(34)
Neutropenia	0.090	0.072	0.108	Beta	(35)
Thrombocytopenia	0.108	0.086	0.130	Beta	(32)
Hypokalemia	0.000	0.000	0.000	Beta	(28)
Fatigue	0.074	0.059	0.088	Beta	(35)
Rash	0.100	0.080	0.120	Beta	(27, 35)
Mucositis	0.441	0.353	0.529	Beta	(28, 33) assumed to be equal to stomatitis
Incidence for treatment related AEs					
TMC group					
Anemia	0.108	0.086	0.130	Beta	(15)
Neutropenia	0.041	0.033	0.049	Beta	(15)
Thrombocytopenia	0.041	0.033	0.049	Beta	(15)
Hyponatremia	0.270	0.216	0.324	Beta	(15)
Fatigue	0.068	0.054	0.082	Beta	(15)
Rash	0.122	0.098	0.146	Beta	(15)
Mucositis	0.054	0.043	0.065	Beta	(15)
TMC-I group					
Anemia	0.171	0.137	0.205	Beta	(15)
Neutropenia	0.066	0.053	0.079	Beta	(15)
Thrombocytopenia	0.053	0.042	0.064	Beta	(15)
Hyponatremia	0.303	0.242	0.364	Beta	(15)
Fatigue	0.092	0.074	0.110	Beta	(15)
Rash	0.092	0.074	0.110	Beta	(15)
Mucositis	0.013	0.010	0.016	Beta	(15)
Discount rate, %	0.003	0.000	0.005	Constant	(18)
Body Surface Area (meters^2^)	1.720	1.380	2.060	Constant	(22)

PFS, progression-free survival; PD, progressed disease; OS, overall survival; AEs, adverse events; TMC, triple metronomic chemotherapy; TMC-I, TMC with intravenous nivolumab **Table 2** summarizes all key model inputs including clinical parameters, cost items, utility values, and AE incidence. Each parameter was associated with a distribution type to enable sensitivity analysis.

¥ = Chinese Yuan (CNY/RMB).

### 2.4 Costs and utilities

According to the Chinese guidelines for pharmacoeconomic evaluation, cost measurement should incorporate all direct healthcare costs as the perspective of the Chinese healthcare system adopted in this study. The cost items included drug costs, genetic testing costs, subsequent treatment costs, disease management costs, best supportive care costs, end-of-life costs, and adverse effect management costs. The prices of nivolumab, methotrexate, celecoxib, and erlotinib were derived from the median price of the drug bidding price in 2023 on YAOZHI database (21). Based on the medication plan and body surface area (22), the per-cycle medication cost for TMC-I was ¥3,270.149, while for the TMC group it was ¥1,420.15. According to the drug instructions of nivolumab (23), patients with head and neck squamous cell carcinoma must undergo a PD-L1 positive assessment using a validated testing method prior to treatment. Therefore, the model includes genetic testing costs, with PD-L1 testing cost derived from the literature (24). Costs related to best supportive care and end-of-life were also obtained from published studies (25, 26). This study only considers grade 3 or above adverse events (AEs) with an incidence rate of 5% or more reported in clinical trials. Management costs for each AE were sourced from published literature (27–30). Details of each cost parameter and the range of values are presented in **Table 1**.

Health-related quality of life was measured using utility scores at a particular health state. Utility scores vary from 1 (indicating perfect utility) to 0 (representing death). The health utility values of each health state were derived from previously published utility study (31). The utility values for the TMC-I group were 0.680 for patients in progression-free survival (PFS) and 0.660 for those in the progressive disease (PD) state. For the TMC group patients, the utility values are 0.610 for PFS status and 0.540 for PD status. AEs-related disutility was extracted from other studies (28, 32–35). All the utility parameters are listed in **Table 2**.

### 2.5 Sensitivity analysis

In this study, we conducted the deterministic sensitivity analysis (DSA) and probabilistic sensitivity analysis (PSA) to assess the robustness of the model outcomes.

In the DSA, each parameter was systematically varied within its predefined plausible range to evaluate the individual impact of each parameter on the model results. The plausible range of each parameter was obtained from published literature or drug bidding price list. When the variation range were not accessible, we assumed that the upper and lower limits fluctuate by 20% around the base value. The plausible ranges are detailed in **Table 2**.

In the PSA, we performed a Monte Carlo simulation consisting of 1,000 iterations. This involved simultaneously sampling the crucial model parameters from appropriate statistical distributions. Costs were assigned a gamma distribution, while the incidence of AEs and utility parameters following a beta distribution. In addition, all parameters in the parametric survival model were assessed through Cholesky decomposition (36). We utilized scatter plots and cost-effectiveness acceptability curves (CEACs) to assess the cost-effectiveness of each treatment regimen across different willingness-to pay thresholds.

### 2.6 Scenario analysis

Given the uncertainty associated with the model assumptions and the sources of parameters in this study, scenario analyses were also performed. In the scenario analysis, we altered the utilities calculated by the progression-based (PB) method to the utilities calculated by the time-to-death (TTD) approach, since different utilities source could yield divergent health-related outcomes (38). The utilities were obtained from a health state utility study estimated TTD-based utility values in HNSCC. The utility were divided according to time to death intervals: ≥183 days (0.694 [95% CI 0.652, 0.736]), 92–182 days (0.651 [95% CI 0.594, 0.707]), 57-91days (0.569 [95%CI 0.484, 0.655]), 29-56days (0.487 [95%CI 0.378, 0.596]), 0-28days (0.422 [95%CI 0.302, 0.541]) (39).

### 2.7 Subgroup analysis

In the subgroup analysis, the ICER was calculated using the subgroup-specific hazard ratios for OS obtained from randomized controlled trial. Since subgroup data for PFS were not available, we assumed that the HRs for PFS of subgroups were the same as for the overall population. We considered the subgroups of patients of different ages, sex, ECOG PS score, Previous treatment (Rx), Previous platinum, Previous taxane, time to failure, and PD-L1 score. Proportional hazards were assumed due to insufficient data.

## 3 Results

### 3.1 Base-case analysis

The results of the base-case analysis are shown in **Table 3**. Over the lifetime period, the total cost of TMC-I regimen was ¥114,585.03, with a utility of 0.76 QALYs, while the total cost of TMC regimen was ¥67,238.04, with a utility of 0.35 QALYs. The TMC-I regimen incurred significantly higher costs compared to TMC, with an additional cost of ¥47,346.98 and a utility gain of 0.41 QALYs. The ICER for TMC-I compared with TMC was ¥116,374.22/QALY. This suggests that TMC-I is cost-effective at a willingness-to-pay threshold of ¥134,037 based on demand-side but not cost-effective at the supply-side threshold of ¥44,679 in China.

**Table 3:** Results of the base-case and scenario analyses.

Base/Scenarios	Intervention	Cost (¥)	QALY	Incremental cost (¥)	Incremental QALY	ICER (¥/QALY)
Base case	TMC	67,238.04	0.35			
	TMC-I	114,585.03	0.76	47,346.98	0.41	116,374.22
Scenario	TMC	67,238.04	0.36			
	TMC-I	114,585.03	0.77	47,346.98	0.41	114,795.25

QALY, quality-adjusted life-year; ICER, incremental cost-effectiveness ratio; TMC, triple metronomic chemotherapy; TMC-I, TMC with intravenous nivolumab.

### 3.2 Scenario analysis

The results of the scenario analysis are also shown in **Table 3**. When using time-to-death (TTD)-based utilities in the scenario analysis, the ICER was ¥114,795.25/QALY. The results remains within the WTP threshold of ¥134,037.

### 3.3 Deterministic sensitivity analysis

**Figure 2:**
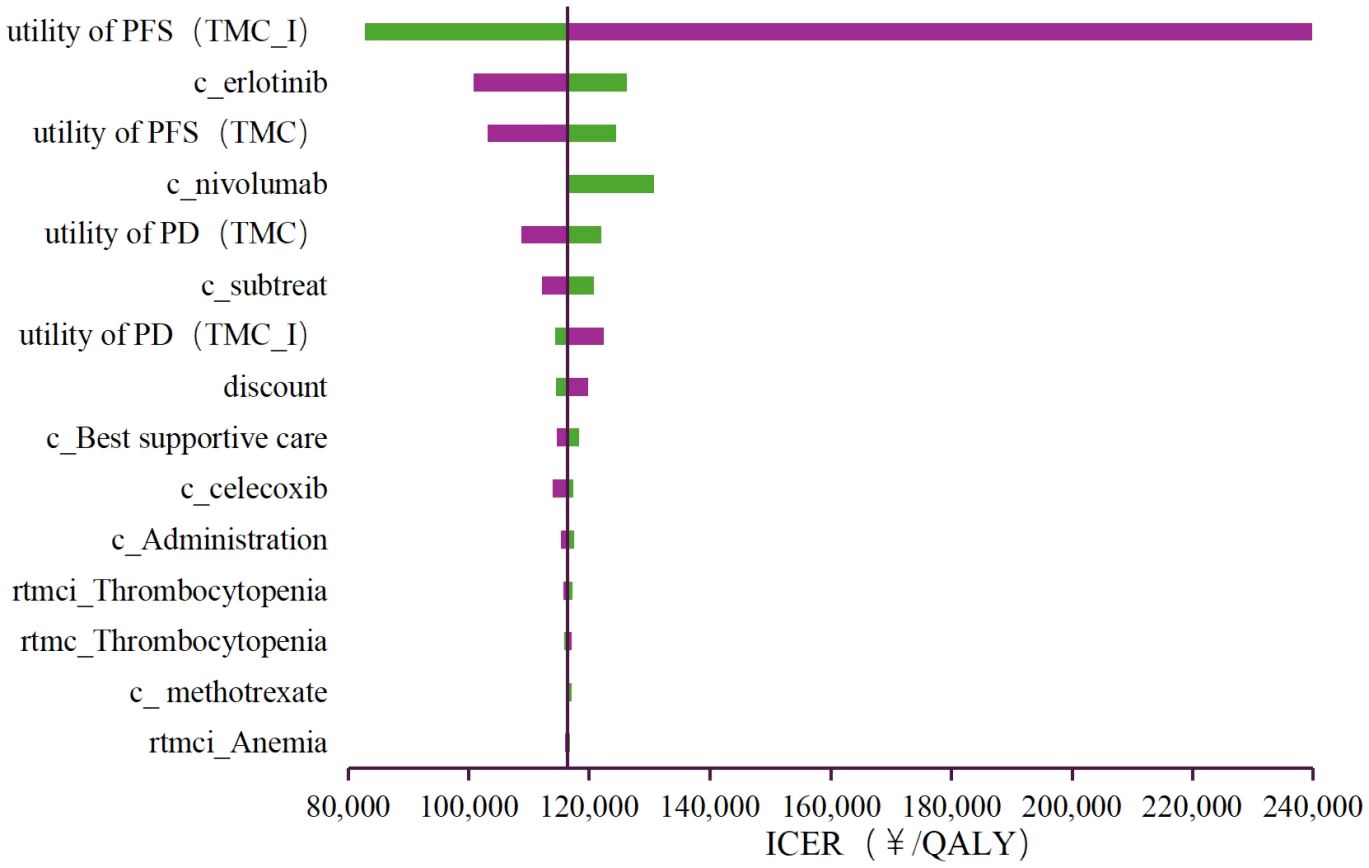
Tornado diagram of the deterministic sensitivity analyses. PFS, progression-free survival; PD, progressed disease; TMC, triple metronomic chemotherapy; TMC-I, triple metronomic chemotherapy with intravenous nivolumab.

Deterministic sensitivity analysis was performed to observe the effect of each parameter variations within the set range on the stability of the results. The findings are illustrated in the tornado diagram (**Figure 2**), which highlights the 15 parameters with the most significant influence on the base-case results. As shown in the figure, the utility of PFS (TMC-I), the cost of erlotinib, and the utility of PFS (TMC) exert a substantial impact on the model results. Among these, the utility of PFS (TMC-I) had the greatest impact on the ICER. When this parameter fluctuates between its upper and lower limits, the ICER ranges from ¥239,986/QALY to ¥281,749.62/QALY, both of which exceed the cost-effectiveness threshold.

### 3.4 Probabilistic sensitivity analysis

**Figure 3:**
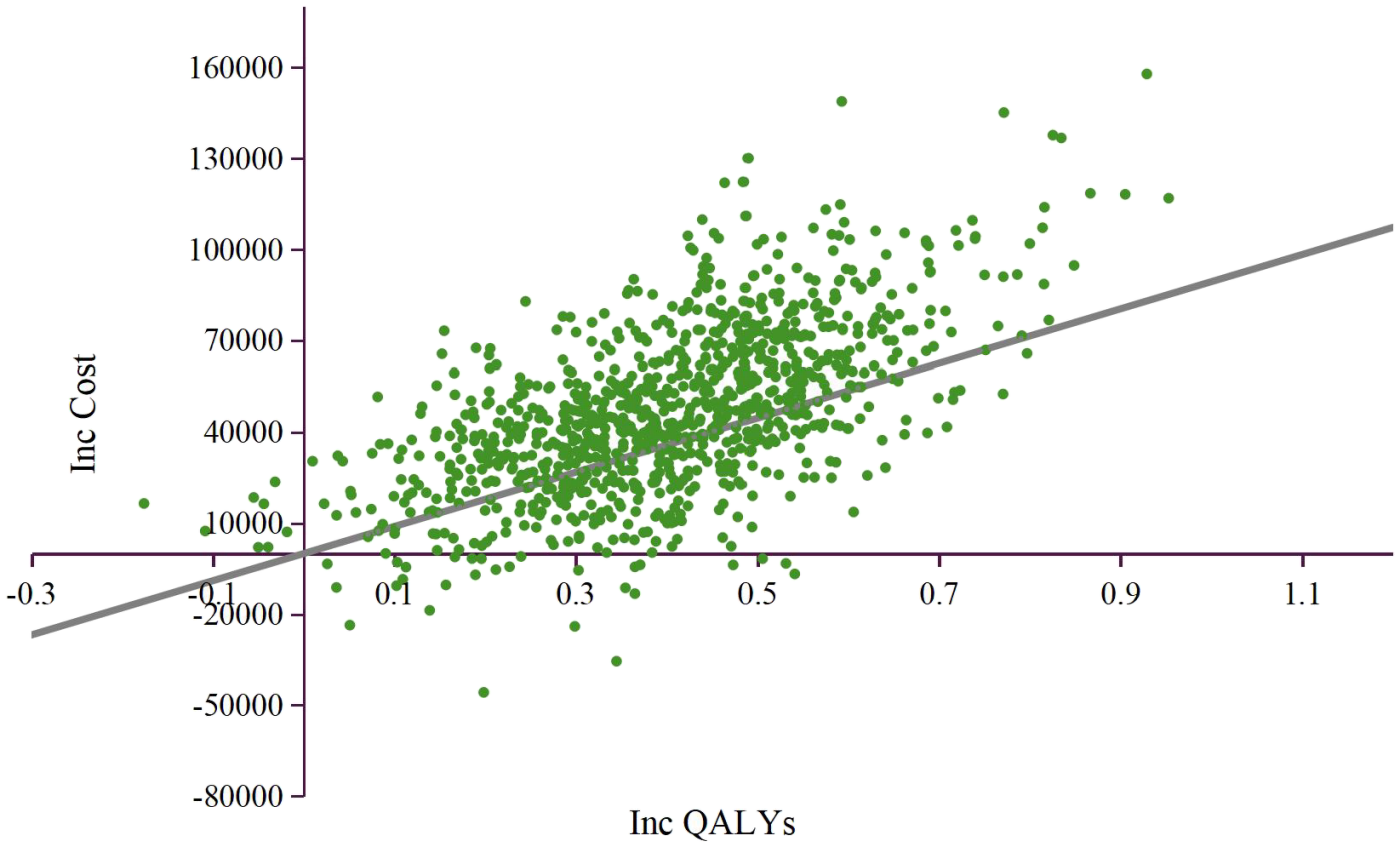
Incremental cost-effectiveness plane. QALY, quality-adjusted life-year; ICER, incremental cost-effectiveness ratio; WTP, willingness to pay.

**Figure 4:**
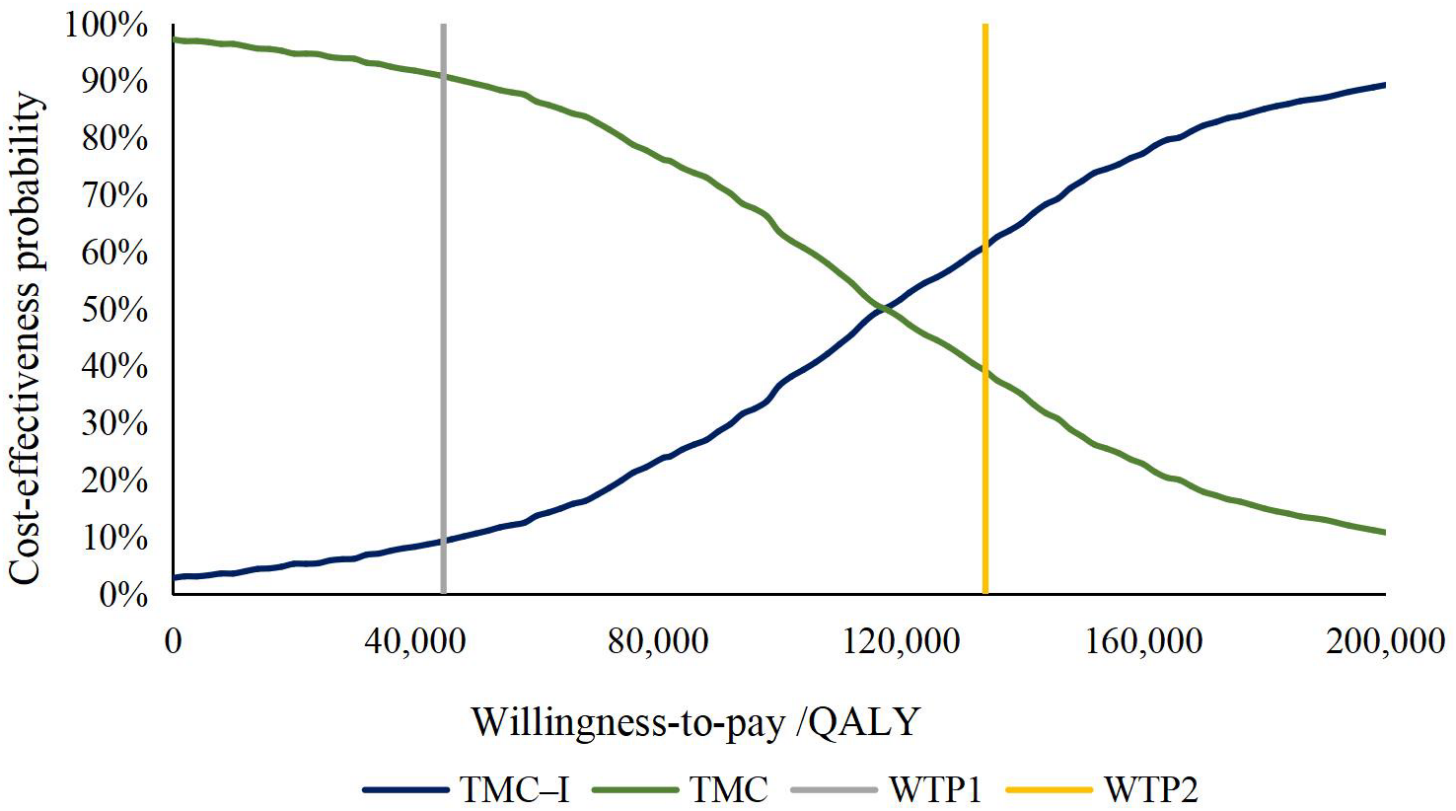
Cost-effectiveness acceptability curve. QALY, quality-adjusted life-year; WTP, willingness to pay.

The results of PSA were showed as the scatter plot in the incremental cost-effectiveness plane (**Figure 3**) and the cost-effectiveness acceptability curves (**Figure 4**). Most of the 1000 simulation results from the PSA fall in the northeast quadrants of the plane, above the WTP threshold. Probabilistic sensitivity analysis comparing the cost-effectiveness of TMC-I vs TMC found that, at a WTP threshold of ¥134,037/QALY, TMC-I had a 60.9% probability of being cost-effective, while at a WTP threshold of ¥44,679/QALY, the probability decreased to 9.4% (**Figure 4**).

### 3.5 Subgroup analysis

The summary results of the subgroup analyses are shown in **Table 4**. The results suggest that TMC-I is cost-effectiveness across all subgroups at a WTP threshold of ¥134,037/QALY. For patients with no previous taxane and PD-L1 score >50, TMC-I was found to be a dominated regime. The TMC-I was also more cost-effective in subgroups including older adult and nonelderly patients, male, ECOG PS =0, ECOG PS =1, prior taxane treatment, and those with PD-L1 score between 1 and 50, compared to the overall population.

**Table 4:** Results of subgroup analysis.

Subgroup	Intervention	Cost (¥)	QALY	Incremental cost (¥)	Incremental QALY	ICER (¥/QALY)
Age, years						
Older Adult	TMC	67,238.04	0.35			
	TMC-I	86,020.73	0.59	18,782.68	0.25	76,292.85
Nonelderly	TMC	67,238.04	0.35			
	TMC-I	75,335.53	0.53	8,097.49	0.18	44,938.20
Sex						
Male	TMC	67,238.04	0.35			
	TMC-I	78,805.19	0.55	11,567.14	0.20	57,173.10
Female	TMC	67,238.04	0.35			
	TMC-I	128,874.76	0.82	61,636.72	0.47	129,789.95
ECOG PS						
0	TMC	67,238.04	0.35			
	TMC-I	109,905.70	0.73	42,667.66	0.38	112,885.46
1	TMC	67,238.04	0.35			
	TMC-I	81,642.22	0.57	14,404.18	0.22	65,512.19
Previous Rx						
Yes	TMC	67,238.04	0.35			
	TMC-I	123,425.17	0.80	56,187.12	0.45	125,575.27
Previous platinum						
Yes	TMC	67,238.04	0.35			
	TMC-I	120,351.82	0.78	53,113.78	0.43	122,994.72
Previous taxane						
Yes	TMC	67,238.04	0.35			
	TMC-I	105,761.59	0.70	38,523.55	0.36	108,170.23
No	TMC	67,238.04	0.35			
	TMC-I	66,484.04	0.47	-754.01	0.12	-6,285.68
Time to failure, months						
<6	TMC	67,238.04	0.35			
	TMC-I	122,005.23	0.79	54,767.18	0.44	124,402.19
PD-L1 score						
>50	TMC	67,238.04	0.35			
	TMC-I	65,905.03	0.46	-1,333.02	0.12	-11,516.68
1-50	TMC	67,238.04	0.35			
	TMC-I	90,501.95	0.62	23,263.90	0.27	85,446.42
10	TMC	67,238.04	0.35			
	TMC-I	114,000.36	0.75	46,762.32	0.40	117,121.12
Unknown	TMC	67,238.04	0.35			
	TMC-I	130,163.16	0.83	62,925.12	0.48	130,725.05

QALY, quality-adjusted life-year; ICER, incremental cost-effectiveness ratio; ECOG PS, Eastern Cooperative Oncology Group performance status; PD-L1, programmed death-ligand 1; Rx, treatment; TMC, triple metronomic chemotherapy; TMC-I, TMC with intravenous nivolumab.

## 4 Discussion

This study evaluates the cost-effectiveness of adding low-dose nivolumab to TMC compared to TMC alone in the treatment of advanced HNSCC patients from the perspective of Chinese healthcare system. According to the RCT, the median OS for TMC and TMC-I was 6.7 and 10.1 months, respectively (hazard ratio, 0.545;95% CI, 0.362 to 0.820;P =.0036). Adding low-dose nivolumab to triple metronomic chemotherapy could improve the overall survival of this patient population. Given its demonstrated efficacy and safety, the combination of low-dose nivolumab and TMC represents a potentially new treatment option for patients unable to access full-dose checkpoint inhibitors, leading to improved prognosis.

In this cost-effectiveness study, TMC-I was found to generate an additional 0.41 QALYs while increasing costs by ¥47,346.98 relative to TMC, leading an ICER of ¥116,374.22/QALY. For Patients with recurrent or newly diagnosed advanced head and neck squamous cell carcinoma, TMC-I was cost-effective at a willingness-to-pay (WTP) threshold of ¥134,037/QALY but not at a threshold of ¥44,679/QALY. The ICER of ¥116,374.22/QALY exceeds the supply-side threshold of ¥44,679, indicating that while TMC-I may be considered cost-effective from a societal perspective, it may not be sustainable under a constrained healthcare budget, especially in under-resourced regions. Sensitivity analysis showed that the utility of PFS had the greatest impact on results. Therefore, the utilities calculated by TTD were adopted in scenario analysis, the results showed that the ICER was ¥114,795.25/QALY, which remained within the WTP threshold of ¥134,037. In the probabilistic sensitivity analysis, the probabilities that TMC-I was cost-effective at thresholds of ¥134,037, ¥44,679/QALY gained were 60.9%, 9.4%, respectively. Subgroup analysis suggested that TMC-I was cost-effective across all subgroups and dominated TMC for patients with no prior taxane treatment and PD-L1 score >50.

To our knowledge, this is the first study to examine the cost effectiveness of low-dose immunotherapy from the perspective of Chinese healthcare system. The economic evidence of nivolumab has been showed in many published literatures (11–13), and remain not a cost-effectiveness regime. Nonetheless, our study demonstrates that low-dose nivolumab may offer a cost-effective alternative. As we all known, Affordability is a critical issue for immunotherapy in low- and middle-income countries, including China, the largest developing country. Introducing and promoting low-dose immunotherapy could therefore have significant implications. Besides, the minimum drug unit size of nivolumab is 40mg/4ml, although low doses are used at a dose of 20 mg every 3 weeks in our study. Evidence suggests that nivolumab remains stable for up to 1 month after opening, making it feasible to use lower doses (40, 41). On the other hand, we should note some limitations of our clinical data sources themselves. Among the entire population, the OS of the TMC-I group was superior to that of the TMC group. However, there was no difference in OS between the TMC group and the TMC group among patients who had not received treatment before. A similar finding was reported in another study (42), with metronomic chemotherapy associated with similar OS compared with intravenous cisplatin in the overall population. However, in the treatment-naïve subgroup, metronomic chemotherapy showed a more significant OS advantage than intravenous cisplatin. This suggests that previously untreated patients may be more likely to benefit from metronomic chemotherapy alone, potentially making the addition of low-dose immunotherapy unnecessary in this subgroup.

This study has several limitations. First of all, due to the lack of local efficacy data and appropriate utility values in China, the clinical data comes from phase III clinical trials of Indian population, the model input utility value came from global population, the heterogeneity of the population may bring certain uncertainties to the results. Although the probabilistic sensitivity analyses of the survival curves were also performed in this study, which has reduced the uncertainty of the clinical data, future low-dose clinical trials in China are necessary. Additionally, refining utility values specifically for Chinese HNSCC patients is recommended. Secondly, uncertainty exists in the prediction of long-term survival for the trial. Health benefits beyond the time of observation of the clinical trial were assumed by fitting parameter distributions to the reported Kaplan-Meier PFS and OS data, which may lead to uncertainty in the model output. Updated data will be needed in the future to validate the results of our model. Thirdly, the dosages and regimens used in this study were based on clinical trials rather than real-world practice. The long-term application of low-dose nivolumab in China will require further evidence and regulatory approval.

Despite these limitations, this study offers valuable insights for patients with HNSCC who may not have access to full-dose checkpoint inhibitors.

## 5 Conclusions

For Patients with HNSCC, adding low-dose nivolumab to triple metronomic chemotherapy is likely to be a cost-effective option from demand-side. These findings provide valuable guidance for physicians and policymakers, aiding clinicians in making informed treatment decisions for HNSCC. The combination of metronomic chemotherapy and immunotherapy holds promise for benefiting an increasing number of patients with advanced cancers.

## Data Availability

The original contributions presented in the study are included in the article/**Supplementary Material**. Further inquiries can be directed to the corresponding author.

## Glossary

HNC: head and neck cancers
HNSCC: head and neck squamous cell carcinoma
CSCO: Chinese Society of Clinical Oncology
PD-1: programmed cell death protein-1
FDA: Food and Drug Administration
TMC-I: adding low-dose nivolumab to triple metronomic chemotherapy
OS: overall survival
CI: confidence interval
TMC: triple metronomic chemotherapy
PSM: partitioned survival model
QALYs: quality-adjusted life years
PFS: progression-free survival
PD: progressive disease
ICERs: incremental cost-effectiveness ratios
WTP: willingness-to-pay
GDP: Chinese gross domestic product
ECOG PS: eastern cooperative oncology group performance status
BSC: best supportive care
KM: Kaplan–Meier
IPD: individual patient data
AIC: Akaike information criterion
BIC: Bayesian information criterion
AEs: adverse events
DSA: deterministic sensitivity analysis
PSA: probabilistic sensitivity analyses
CEACs: cost-effectiveness acceptability curves
PB: progression-based
TTD: time-to-death
Rx: previous treatment
