# Supplementary material for: Cost-effectiveness analysis of the combination of low-dose nivolumab with triple metronomic chemotherapy for advanced head and neck squamous cell carcinoma in China
Source: Front Oncol. 2025 Sep 1;15:1542792. doi: 10.3389/fonc.2025.1542792 (PMC12433840; doi:10.3389/fonc.2025.1542792)
Supplement: Supplementary file 1 [file DataSheet1.docx]

**Cost-effectiveness analysis of the combination of low-dose nivolumab with triple metronomic chemotherapy for advanced head and neck squamous cell carcinoma in China**

Yingdan Cao^1,2 *^, Fenghao Shi ^3 *^, Xiaoxia Wei^4^ , Sheng Han ^3^, Yu Fang^1,2 #^

^1^ Department of Pharmacy Administration and Clinical Pharmacy, School of Pharmacy, Xi’an Jiaotong University, Xi'an, China,

^2^ Center for Drug Safety and Policy Research, Xi’an Jiaotong University, Xi'an, China,

^3^ International Research Center for Medicinal Administration, Peking University, Beijing, China,

^4^ Department of Pharmacy, Shengli Clinical Medical College of Fujian Medical University, Fujian Provincial Hospital, Fuzhou, China

SUPPLEMENTARY MATERIALS

# FIGURE S1. The reconstruction of Kaplan–Meier curves

Reconstructed Kaplan‐Meier curves of overall survival (OS) and progression-free survival (PFS) pooled from a randomized clinical trial, for patients with advanced head and neck squamous cell carcinoma who received low-dose nivolumab with triple metronomic chemotherapy (TMC-I) or triple metronomic chemotherapy alone (TMC) (Figures S1-1 and S1-2).

**
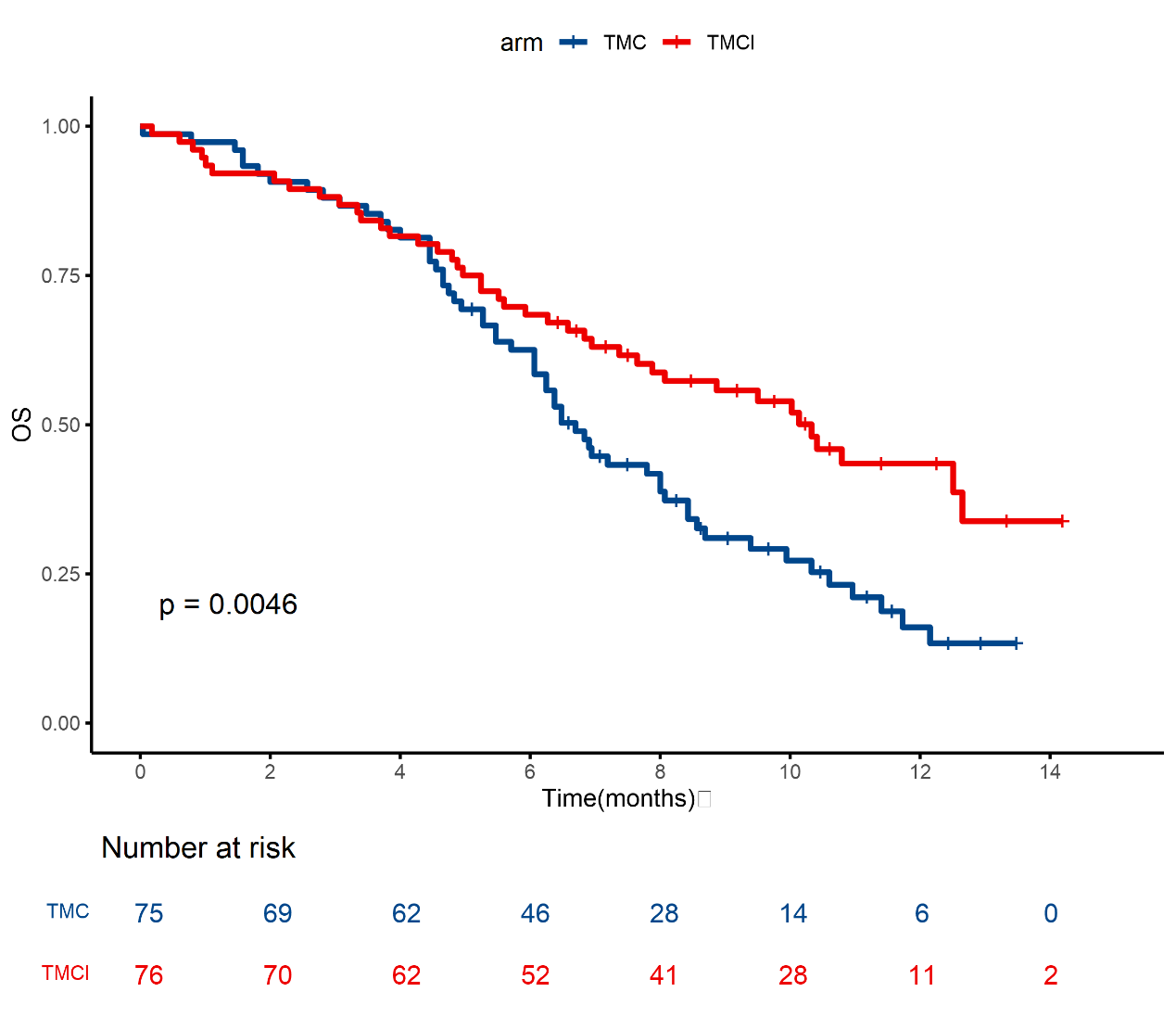
**

**Figure S1-1. Reconstructed Kaplan–Meier curves of overall survival**

**
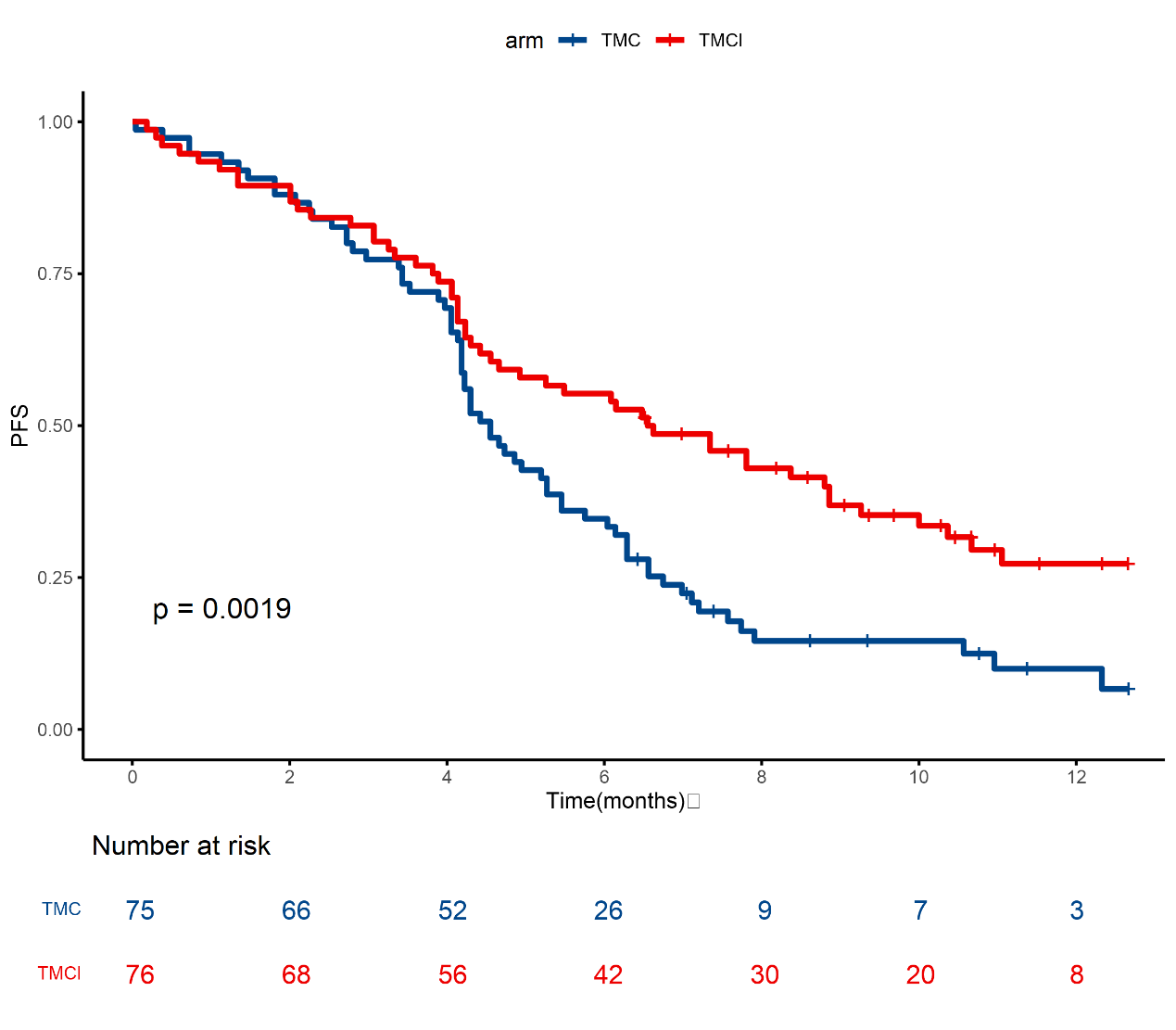
**

**Figure S1-2. Reconstructed Kaplan–Meier curves of progression-free survival**

# FIGURE S2. The selection of survival models

Appropriate survival models can be selected by comparing log cumulative hazard plots for overall survival (OS) and progression-free survival (PFS) between low-dose nivolumab with triple metronomic chemotherapy (TMC-I) or triple metronomic chemotherapy alone (TMC) (Figures S2-1 and S2-2). The plots of the two groups were not parallel, suggesting that the proportional hazards assumption may not hold and that each treatment arm should be analyzed separately.


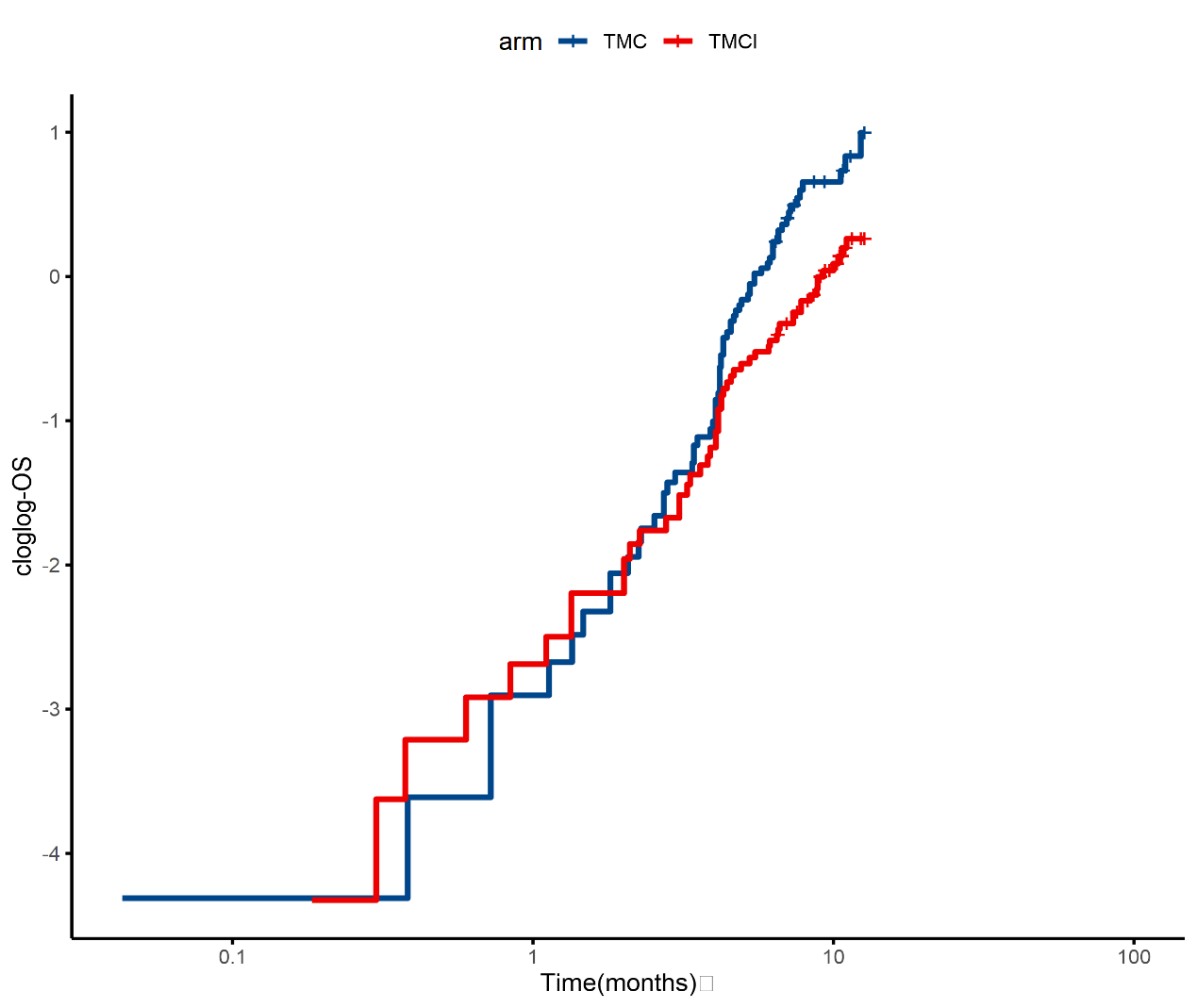


**Figure S2-1. Log cumulative hazard plots of overall survival**


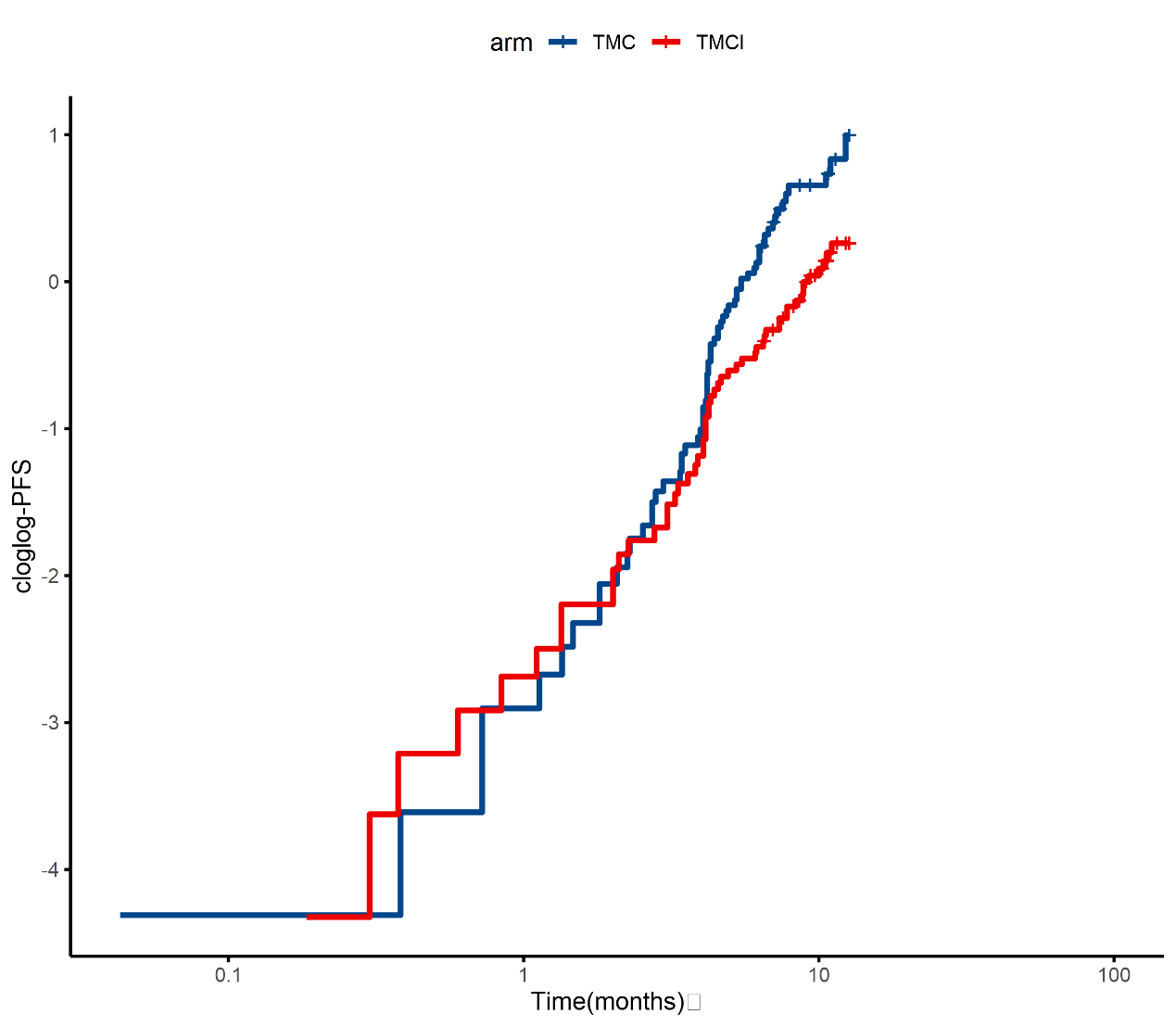


**Figure S2-2. Log cumulative hazard plots of progression free survival**

# FIGURE S3. The fitting and extrapolation of Kaplan-Meier curves for OS and PFS

The fitting and extrapolation of Kaplan-Meier curves for OS and PFS were based on common parametric survival models, such as exponential, gamma, Gompertz, Weibull, log-logistic, and log-normal, and the most appropriate survival function was selected based on statistical fit using Bayesian Information Criterion (BIC) and Akaike Information Criterion (AIC).

## *OS Fit*

(1) Low-dose nivolumab with triple metronomic chemotherapy (TMC-I)

As for the OS Kaplan-Meier curve of TMC-I, the statistical fits of six parametric survival models are presented in Figure S3-1 and Table 1. The exponential distribution had the lowest BIC, it may be appropriate for OS of TMC-I. Therefore, the exponential distribution was selected in our study.


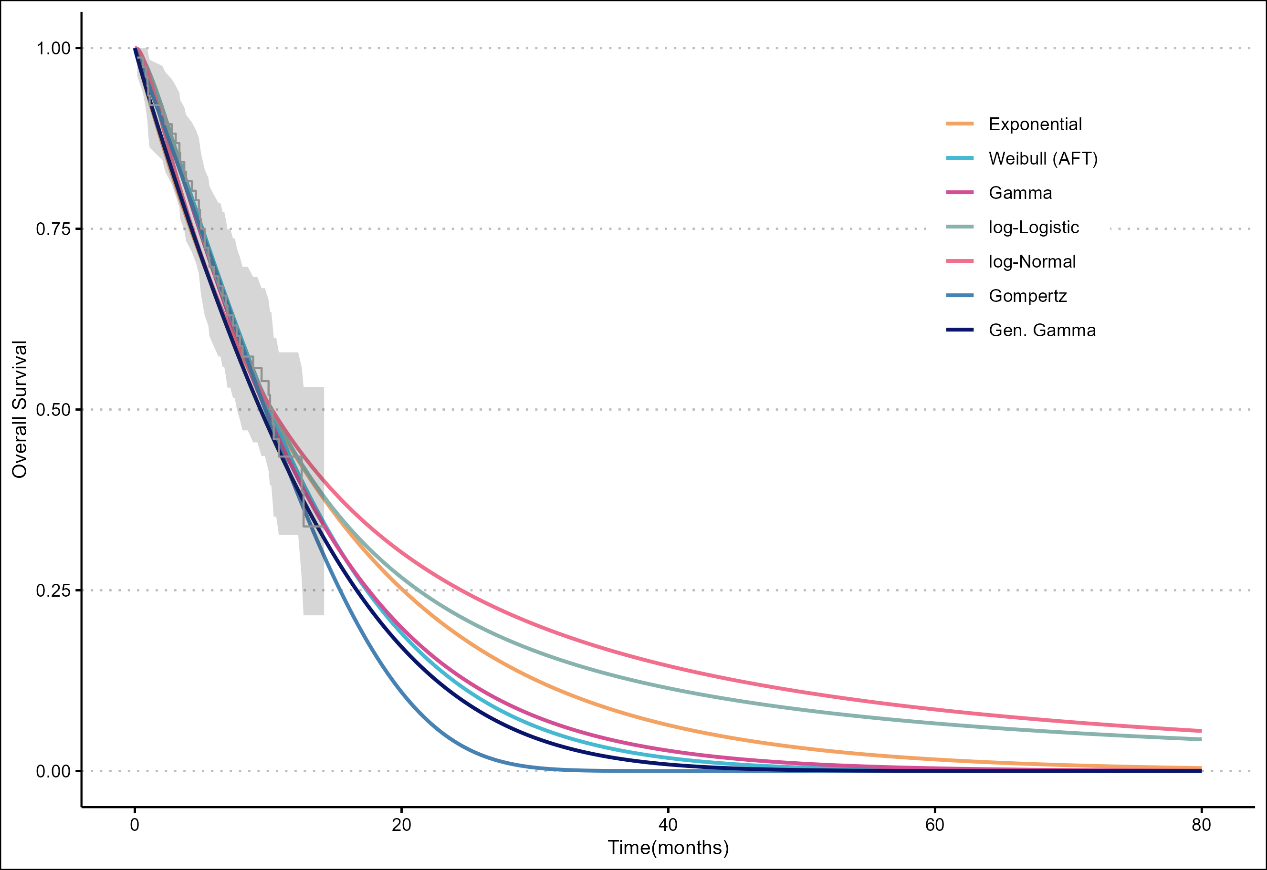


**Figure S3-1. Six parametric survival models for the OS Kaplan-Meier curve of TMC-I group**

1. Triple metronomic chemotherapy alone (TMC)

As for the OS Kaplan-Meier curve of TMC, the statistical fits of six parametric survival models are presented in Figure S3-2 and Table 1. The weibull distribution had the lowest AIC and BIC, it may be appropriate for OS of TMC. Therefore, the weibull distribution was selected in our study.


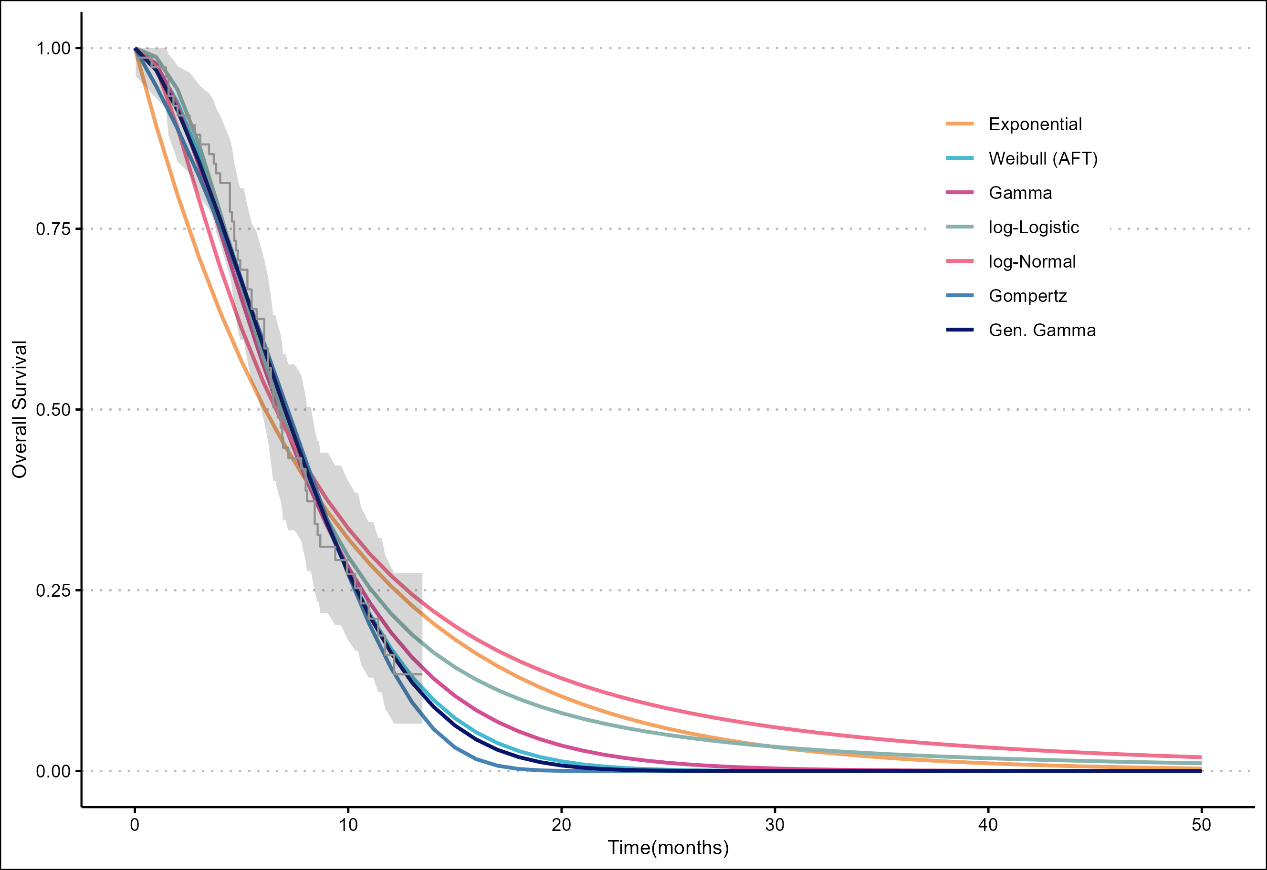


**Figure S3-2. Six parametric survival models for the OS Kaplan-Meier curve of TMC group**

## *PFS Fit*

1. Low-dose nivolumab with triple metronomic chemotherapy (TMC-I)

As for the PFS Kaplan-Meier curve of low-dose nivolumab with triple metronomic chemotherapy, the statistical fits of six parametric survival models are presented in Figure S3-3 and Table 1. The Exponential distribution had the lowest BIC, it may be appropriate for PFS of TMC-I. Therefore, the Exponential distribution was selected in our study.


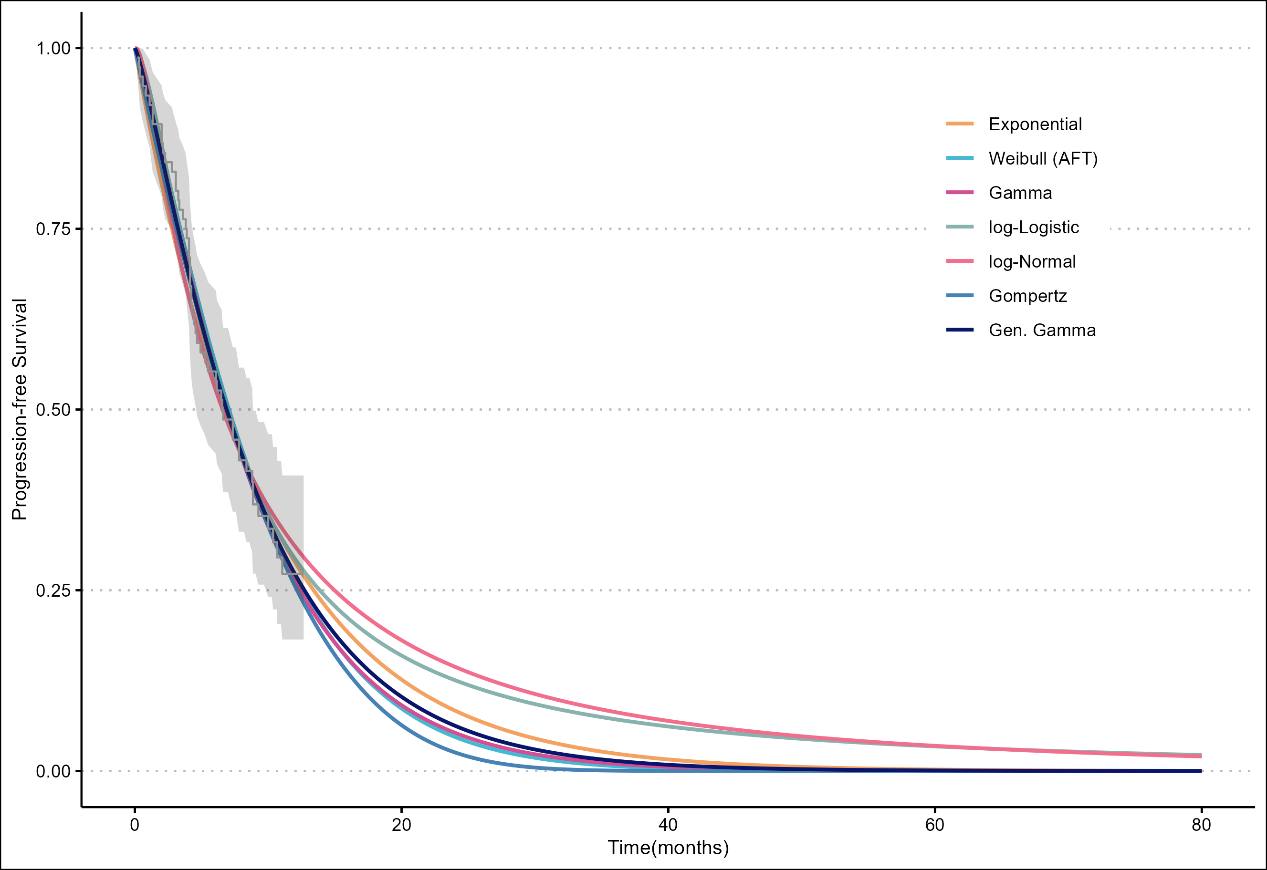


**Figure S3-3. Six parametric survival models for the PFS Kaplan-Meier curve of TMC-I group**

1. Triple metronomic chemotherapy (TMC)

As for the PFS Kaplan-Meier curve of TMC, the statistical fits of six parametric survival models are presented in Figure S3-4 and Table 1. The Weibull distribution had the lowest AIC and BIC, it may be appropriate for PFS of TMC. Therefore, the Weibull distribution was selected in our study.


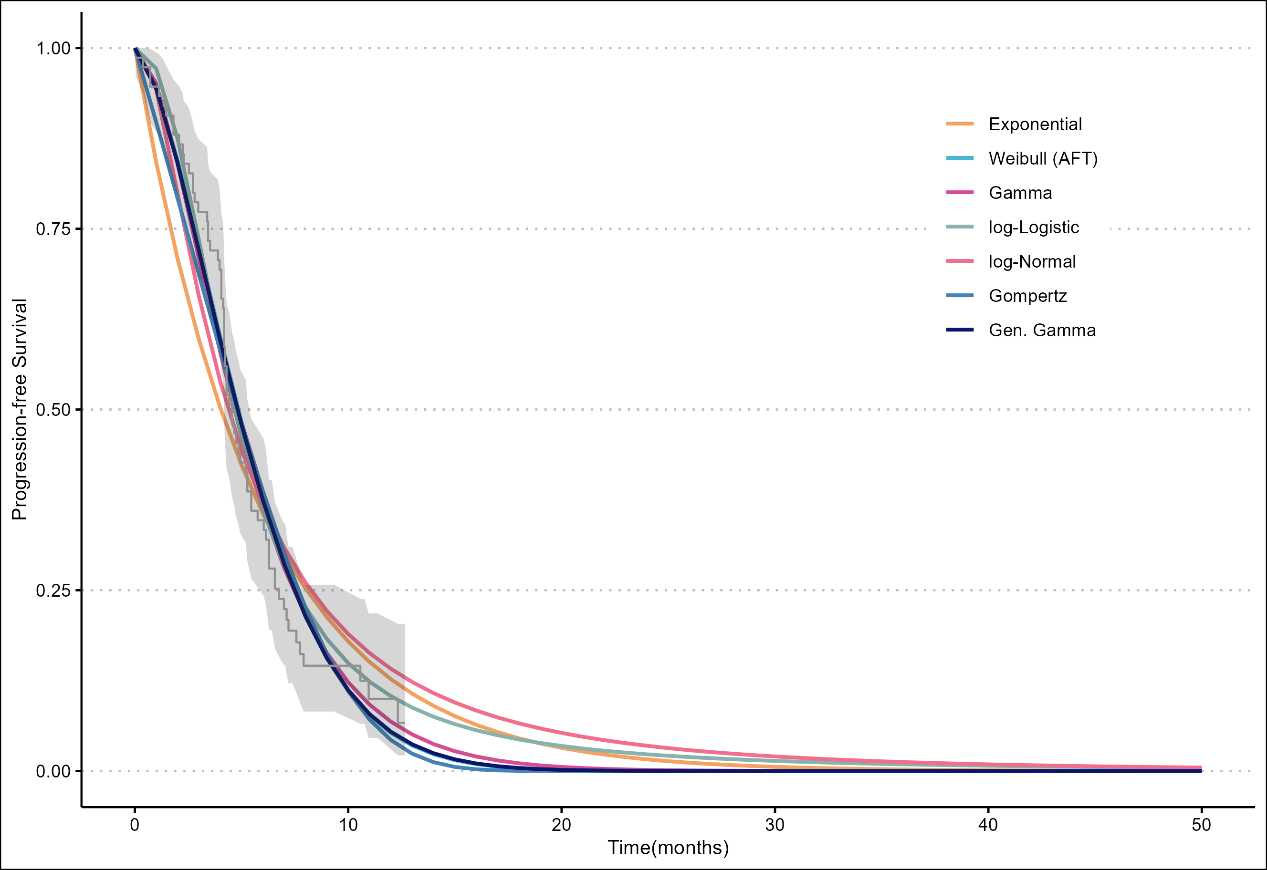


**Figure S3-4. Six parametric survival models for the PFS Kaplan-Meier curve of TMC group**
